# The mechanisms of nerve injury caused by viral infection in the occurrence of gastrointestinal motility disorder-related diseases

**DOI:** 10.1186/s12985-023-02185-x

**Published:** 2023-11-01

**Authors:** Yaqian Li, Qiuyu Chen, Liwei Wang, Xin Chen, Bangmao Wang, Weilong Zhong

**Affiliations:** 1 Department of Gastroenterology and Hepatology, Tianjin Institute of Digestive Diseases, Tianjin Key Laboratory of Digestive Diseases, Tianjin Medical University General Hospital, Tianjin, 300052 China; 2 Department of Gastroenterology, Tianjin First Central Hospital, Tianjin, 300110 China

**Keywords:** Gastrointestinal motility, Viral infection, Enteric nervous system

## Abstract

Gastrointestinal motility refers to the peristalsis and contractility of gastrointestinal muscles, including the force and frequency of gastrointestinal muscle contraction. Gastrointestinal motility maintains the normal digestive function of the human body and is a critical component of the physiological function of the digestive tract. At present, gastrointestinal motility disorder-related diseases are gradually affecting human production and life. In recent years, it has been consistently reported that the enteric nervous system has a coordinating and controlling role in gastrointestinal motility. Motility disorders are closely related to functional or anatomical changes in the gastrointestinal nervous system. At the same time, some viral infections, such as herpes simplex virus and varicella-zoster virus infections, can cause damage to the gastrointestinal nervous system. Therefore, this paper describes the mechanisms of viral infection in the gastrointestinal nervous system and the associated clinical manifestations. Studies have indicated that the means by which viruses can cause the infection of the enteric nervous system are various, including retrograde transport, hematogenous transmission and centrifugal transmission from the central nervous system. When viruses infect the enteric nervous system, they can cause clinical symptoms, such as abdominal pain, abdominal distension, early satiation, belching, diarrhea, and constipation, by recruiting macrophages, lymphocytes and neutrophils and regulating intestinal microbes. The findings of several case‒control studies suggest that viruses are the cause of some gastrointestinal motility disorders. It is concluded that one of the causes of gastrointestinal motility disorders is viral infection of the enteric nervous system. In such disorders, the relationships between viruses and nerves remain to be studied more deeply. Further studies are necessary to evaluate whether prophylactic antiviral therapy is feasible in gastrointestinal motility disorders.

## Introduction

**Fig. 1:**
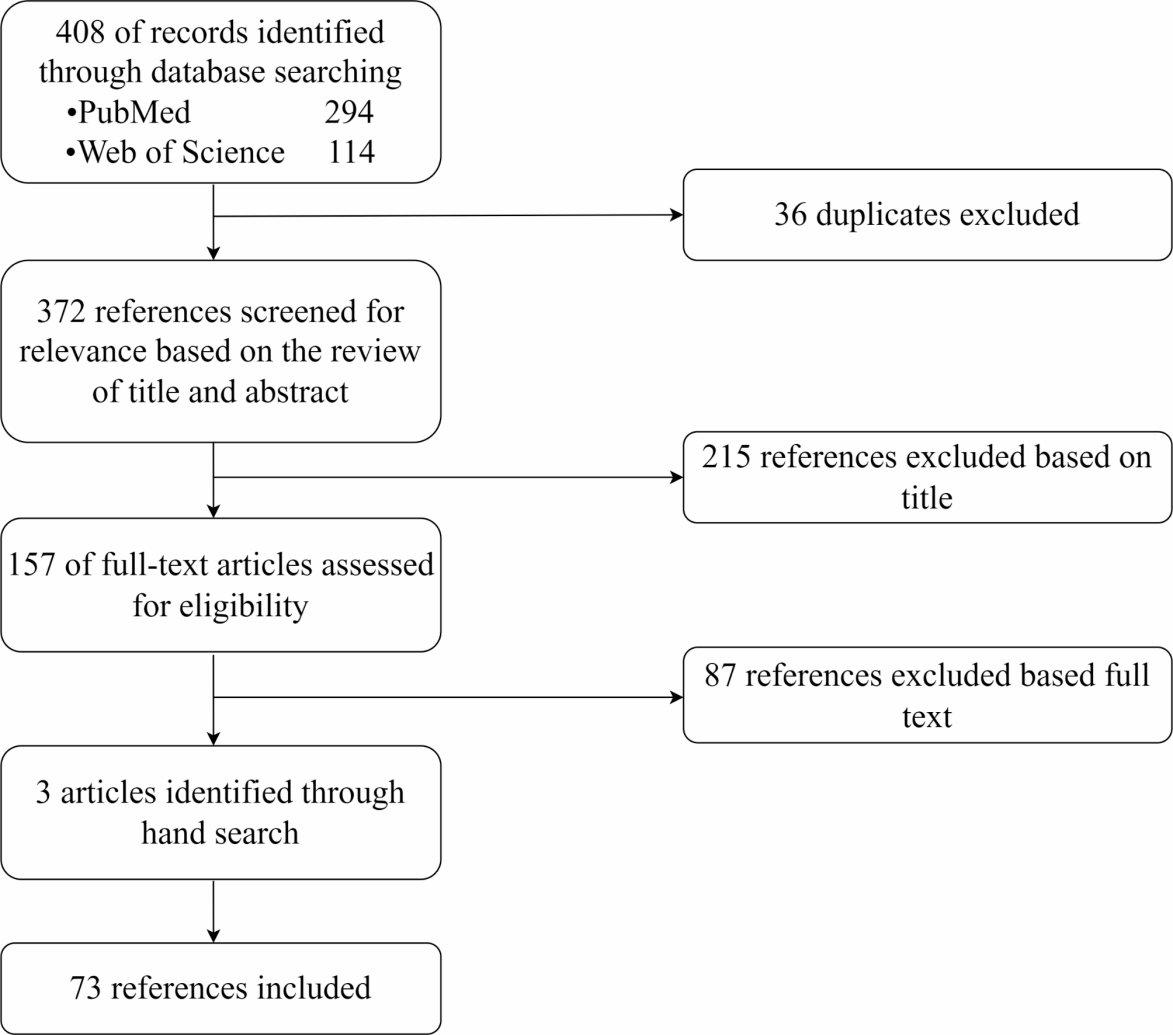
Literature search flow chart

At present, gastrointestinal (GI) motility disorders are motor or sensory disorders of the gastrointestinal tract caused by disorders in the regulation of nerve innervation, such as achalasia, esophageal reflux disease and intestinal pseudo-obstruction [1–4]. However, there are other diseases, such as functional gastrointestinal disorders and some systemic diseases, that are also accompanied by GI motility abnormalities [5, 6]. These GI motility disorders affect a large proportion of the population and affect their quality of life [7]. The prevalence of functional dyspepsia is approximately 16% worldwide [8]. As a kind of esophageal motility disorder, the prevalence of achalasia is as high as 12.6/100,000 [9]. The motor function of the GI tract depends on the GI nervous system [10]. Motility dysfunction caused by functional or anatomical changes in the GI nervous system is one of the main factors contributing to these diseases. Some neurotropic viruses can infect the GI nervous system and cause enteric neuromuscular dysfunction along with nerve damage [11]. Among them, the rate of varicella zoster virus infection has been reported to be essentially higher in patients with achalasia than in controls [12]. The digestive system of vertebrates has a very complex neural network. First, as with most systems, the digestive system receives extrinsic neuronal innervation from autonomic nerves, including sensory nerve fibers from the vagus ganglia and dorsal root ganglia in the spinal cord [13]. Second, the digestive system has its own unique intrinsic nervous system, namely, the enteric nervous system (ENS) [14–16]. The ENS is the largest and most complex part of the peripheral nervous system [17, 18] and consists of the neurons and glia in two major plexuses: the myenteric plexus and the submucosal plexus [19]. This article provides a systematic review of the literature on the mechanism of neurological damage caused by viruses in GI motility disorders. The literature search process cited in this paper is shown in Fig. 1.

## Viral infection of the intestinal ganglia

At present, there is evidence that the viruses that infect the ENS are mainly herpes simplex virus (HSV) and varicella-zoster virus (VZV), and the novel coronavirus that has become a global pandemic since 2019 can also infect the ENS [20–22]. Latent infection, lytic infection, and reactivation occur when viruses, such as VZV, reach enteric neurons through various pathways [23–25]. The distinction between lytic and latent infection can be determined by the presence or absence of cell-associated viral infection [26, 27]. Latent infection allows the virus to be transcribed and translated in neurons but does not produce any symptoms. In vitro, neurons establish latent infection when neurons are exposed to isolated VZV without associated cellularity. Many studies show that they also express mRNAs encoding ORFs 4, 21, 29, 40, 62, and 63, with ORF 62 and 29 proteins in the cytoplasm [28]. During latent infection, latent genome replication is not completely turned off, and there are several viral genes that accumulate in neurons in addition to latent-associated transcripts [29]. In contrast, in lytic infection, neurons die within 48 hours when exposed to nonneuronal cell-associated VZV. During this period, neurons express gB, gE, ORF 62p and ORF 29p. ORF 62 and ORF 29 proteins are located in the nucleus when lytic infection occurs [28].

Additionally, infections of the enteric nervous system may be associated with vaccine administration. Studies have shown that either through natural infection or through varicella vaccination, almost everyone exposed to VZV acquires latent VZV in the ENS [12, 26].

## Viral infection-induced damage to neural pathways

**Fig. 2:**
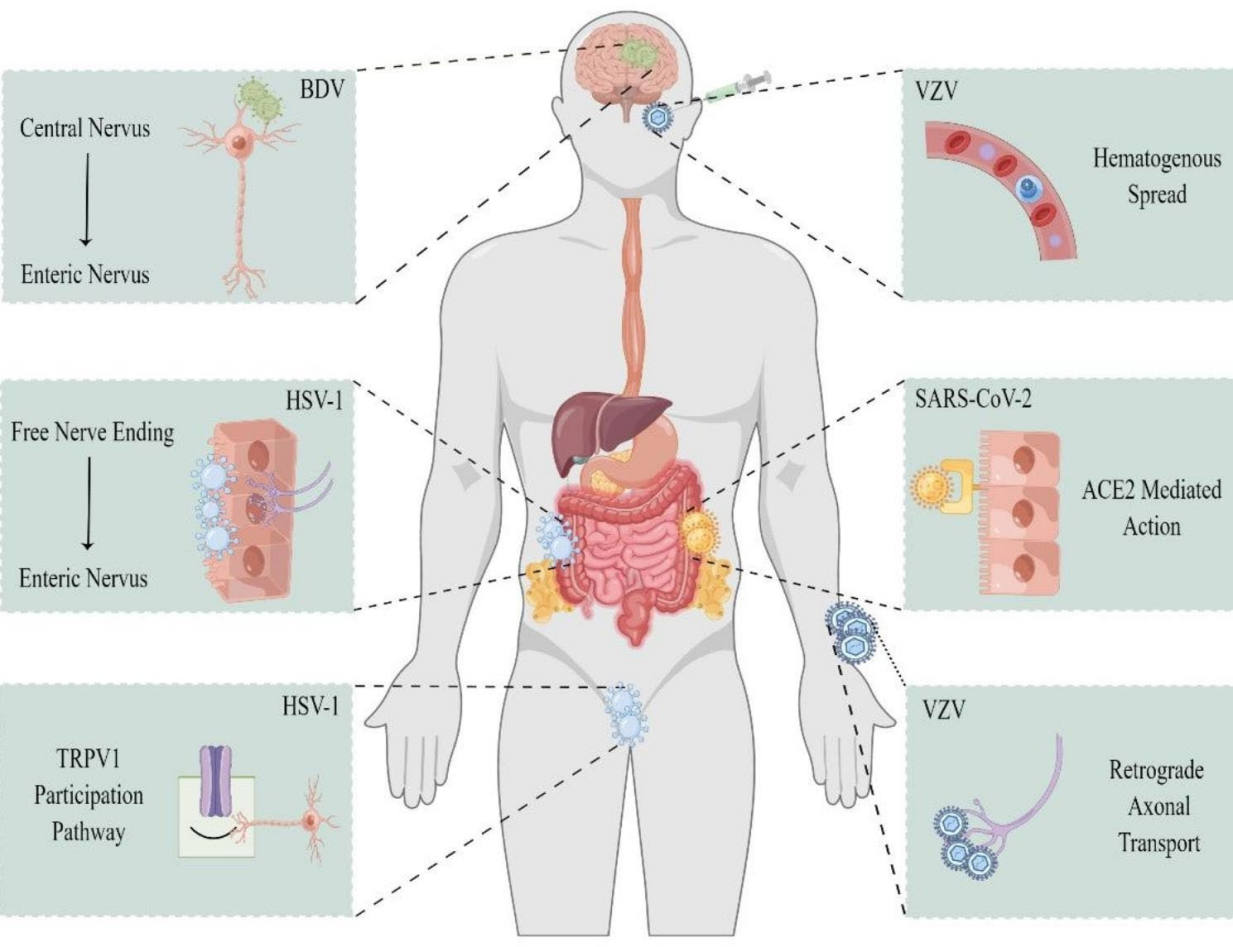
Mechanisms by which various viruses infect and spread to the ENS

To some extent, viruses can become pathogenic by infecting nerves and damaging neurons. For GI motility diseases, the virus invades the nerve and causes damage in several ways, including retrograde axonal transport after the infection of nerve endings, hematopoietic spread after the infection of lymphocytes, and the involvement of nociceptors or ACE2 receptors (Fig. 2).

### Axonal transport of viruses

#### Transmission from the skin surface

**Fig. 3:**
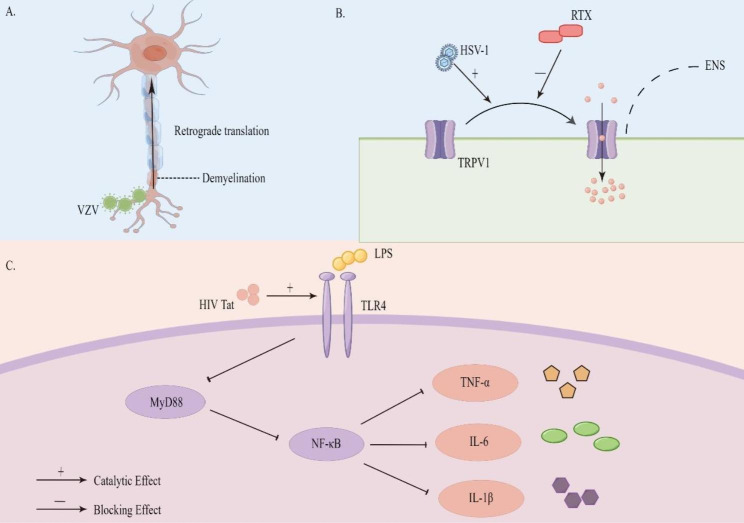
Some specific mechanisms of viral infection. **A**. VZV infects unmyelinated sensory nerve endings and is transported in a retrograde manner. **B**. RTX prevents HSV-1 entry into the ENS by disrupting peripheral TRPV1 nociceptors. **C**. HIV Tat promotes the LPS-TLR4-MyD88-NF-κB pathway through TLR4, thereby promoting the release of inflammatory factors

Varicella-zoster virus is a type of neurotropic virus [30]. Some experiments in which the skin and guts of animals are double-labeled with retrograde tracers have shown that the virus can be transported retrogradely along nerve fibers [26, 29]. Sensory nerves innervating the skin become demyelinated within the epidermis. In a varicella attack, when the epidermis is infected with VZV, the unmyelinated sensory nerves under the area of VZV-infected skin become infected with cell-free virions [31]. After infection, cell-free virions propagate in a retrograde manner along sensory pathways to the dorsal root ganglion (DRG). In addition, a subset of DRG neurons send nerve fibers that meander to the intestine and directly infect the ENS to form latent infections (Fig. 3A). The virus is transported through the axons of the sensory neurons of the skin epidermis and then transferred to the ENS, leading to latent infection. When the body is reinfected with VZV, intestinal motility disorders occur [26, 32, 33].

#### Transmission from the intestinal mucosa

HSV-1 has been confirmed to form latent infection after oral inoculation into the lumen of mice [29, 34–36]. A study has shown that the intestinal epithelium contains immune-related cells, such as M cells, that absorb and transport viral antigens. Moreover, HSV-1 may cross intestinal epithelial cells through specific receptor-mediated endocytosis and destruction of tight junctions between cells [37]. The free nerve endings of the ENS can directly interact with surface epithelial cells. Through these mechanisms, the virus can enter the ENS directly through nerve endings. Alternatively, the simultaneous observation of uninfected and highly infected neurons in the same ganglion proves that intraganglionic transmission is most likely through specific transneuronal transmission rather than between neighboring cells [34]. Aujeszky’s disease virus (ADV) is a neurotropic herpesvirus that occurs in young pigs. In an experiment in which ADV was injected into the duodenal lumen of piglets, necrotizing enteritis and myoenteritis developed several weeks later. Intestinal pathological and immunohistochemical analyses supported that ADV invaded the myenteric plexus from the intestinal lumen and spread through the solar plexus and ganglion [38].

#### Centrifugal propagation from the CNS

Neurotropic viruses may infect the central nervous system (CNS) and then be transmitted to the ENS through centrifugal propagation. Borna disease virus (BDV) is a neurotropic agent that can infect a wide range of warm-blooded species. It has been reported that BDV immunoreactivity can be detected from neurons and nerve fibers in the submucosal plexus as well as the myenteric plexus of infected mice 4–14 weeks after intracerebral BDV infection. During this process, the expression of Calbindin D-28k (CALB) was found to be upregulated in the myenteric plexus neurons of infected mice compared with uninfected mice. Therefore, we reasoned that CALB expression might be induced after BDV infection of mice. Additionally, submucosal neurons were determined to be more highly BDV immunoreactive than intramuscular neurons. There are two presumed reasons for the different degrees of response: first, the degree of innervation of the submucosal and intramuscular plexuses by exogenous nerve fibers is different. Second, the infectivity of the two plexuses to BDV is different [39].

### Hematogenous dissemination to invade neurons

VZV can infect T lymphocytes. Chen et al. performed experiments in which peripheral blood mononuclear cells (PBMCs) from humans and guinea pigs were isolated in vitro and cocultured with human embryonic lung fibroblasts (HELFs) infected with VZV and fluorescent agents. After a while PBMCs were infected with VZV. The fluorescence of the virus was consistent with the immunofluorescence of CD3, which confirmed that the infected cells were T lymphocytes. The appearance of enteric neuronal infection in guinea pigs 2 days after intraorbital sinus injection of infected T lymphocytes suggested that infected T lymphocytes transmit the virus to enteric neurons via blood transmission. After subcutaneous injection of cell-associated VZV, VZV DNA and transcripts were found in blood leukocytes, indicating the possibility of virus transmission to enteric neurons through blood [26]. Gan et al. similarly transferred VZV to guinea pig PBMCs by a coculture approach. T lymphocytes in PBMCs were preferentially infected with VZV after coculture with VZV-infected HELFs. Twenty-eight days after intravenous injection of infected PBMCs into guinea pigs, hybrid VZV DNA was detected in the myenteric plexus ganglia, and the ORF 63p immunoreactive cells all showed immunoreactivity for the neural marker HuC/D. These results suggested that VZV infected the intestinal neurons of guinea pigs and that latent infection was established [27]. However, based on previous analyses, cell-associated VZV infection of neurons causes lytic infection. Therefore, we speculate that infected T cells secrete isolated cell-free VZV that infects enteric neurons to lead to latent infection; alternatively, this can occur owing to the viremia resulting from VZV infection.

### Other types of transmission

A mouse model of genital HSV-1 infection was studied by Khoury-Hanold et al. This model did not fully demonstrate the transmission pathway from genital infection to the development of fatal toxic megacolon. However, the researchers observed that HSV-1 in the mouse model must first infect sensory neurons and then spread to the bladder, paracervical ganglion and DRG within 12 days. Subsequently, they used resiniferatoxin (RTX) to specifically bind to transient receptor potential cation channel subfamily V member 1 (TRPV1) to eliminate the nociceptive receptor. The results revealed that RTX treatment prevented the virus from reaching the DRG and was effective in preventing eventual GI motility disorders (Fig. 3B). Therefore, peripheral TRPV1 nociceptors are involved in the pathway of viral transmission to the ENS [40].

In addition, VZV infects tonsil T cells after inhalation and mediates the transport of T cells to the skin, causing epidermal destruction [41, 42]. Sen et al. cocultured ${CD3}^{+}$ T cells from tonsils with infected HELFs to infect T cells. By comparison, the infected T cells expressed unique phenotypic characteristics, and VZV induced changes in the surface proteins of T cells. Among them, ${CCR7}^{-}{CD27}^{-}{CD127}^{-}$indicated the presence of tissue homing and effector phenotypes. Otherwise, enhanced expression of CD11a and CD49d promoted the activation and mediated the transendothelial migration of T cells. Furthermore, some T cells expressed CCR4 and CLA, hallmark features of cutaneous trafficking. VZV affects at least two important cellular signaling pathways: the initiation of the Zap70 cascade and the activation of the Akt pathway. Both can enhance the survival and migration of T cells [43].

SARS-CoV-2 is the causative agent of COVID-19. It infects the GI tract by invading host cells through ACE2 receptors. The spike glycoprotein binding domain on the surface of the virus has high affinity and can be cleaved by endogenous proteases such as Furin and TMPRSS2 before binding, allowing more efficient binding to the ACE2 receptor on the host cell surface [44]. After binding, the virus and ACE2 receptor are endocytosed into the host cell, which in turn initiates virus replication, secretion and release. The ACE2 receptor is expressed in multiple organs, and its expression is highest in the brush border of the intestinal epithelium. A study detected that ACE2 and TMPRSS2 are abundantly expressed in the perinuclear region of enteric neurons and glial cells in the myenteric and submucosal plexus after SARS-CoV-2 infection, which reasonably proves viral infection of enteric neurons. At present, it is believed that the ENS may be a potential route for SARS-CoV-2 to enter the brain [22, 45–48].

## Gastrointestinal dysfunction due to viral infection of nerves

**Fig. 4:**
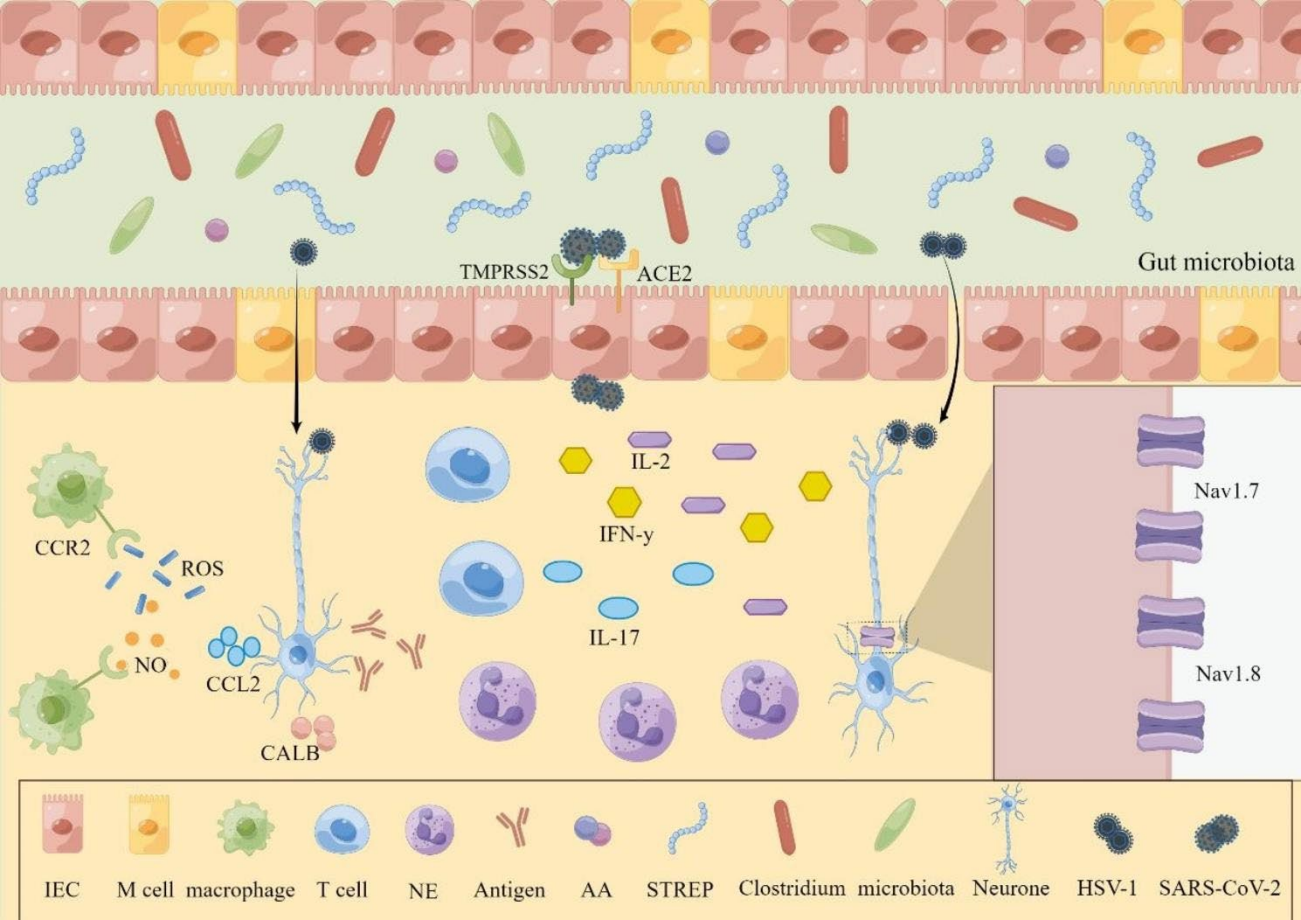
Gastrointestinal responses following viral infection of the ENS. After entering the GI tract, different viruses pass through the intestinal mucosa through their own means. HSV-1 is directly taken up and transported by intestinal epithelial cells and then enters the ENS axon terminals. Alternatively, it crosses intestinal epithelial cells by disrupting tight junctions between cells. SARS-CoV-2 is transported through the ACE2 receptor, and TMPRSS2 can enable the virus to bind to the receptor more efficiently. Following viral infection, a range of responses occur. CCL2 released by the ENS recruits macrophages through the CCL2/CCR2 pathway. The recruitment of various inflammatory cells leads to the release of inflammatory factors. There are also changes in gut microbiota and the expression of sodium channel isoforms

It is well known that the gastrointestinal nervous system regulates gastrointestinal motility [49]. When the virus is latent in neurons, there are no obvious symptoms in the digestive tract. However, when the body is re-infected, the latent infection can be reactivated [26] and lead to symptomatic or asymptomatic infection of the gastrointestinal tract through various mechanisms or reactions [11, 29, 50] (Fig. 4).

### Macrophages recruitment through the CCL2/CCR2 pathway

HSV-1 is a common neurotropic virus that can be latent in the body’s ENS [11]. One study developed a mouse model of ENS infection with HSV-1 by gavage inoculation with HSV-1. Infected mice had more rapid gastric emptying, delayed intestinal transit, slower colonic motility [11, 29], and transient impairment in intestinal neuromuscular contractility compared with mice in the sham-infected group. It was further revealed that CD11${b}^{+}$F4/${80}^{+}$ cells were barely detectable in the longitudinal myenteric plexus (LMMP) of sham-infected mice, whereas the infected group of mice showed an increase in CD11${b}^{+}$F4/${80}^{+}$ macrophages from the first week onwards. In addition, after gavage inoculation, HSV-1 infection increased the expression of neuronal nitric oxide synthase protein in LMMP neurons, and activated macrophages produced free radicals, namely, reactive oxygen species and nitric oxide. The gastrointestinal motility of HSV-1-infected mice was significantly improved after the administration of the nonselective NOS inhibitor L-NAME, the selective iNOS inhibitor AR-C102222, and clodronate-containing liposomes. At the same time, the study treated HSV-1-infected mice with RS504393, a highly selective CCR2 chemokine receptor antagonist. Immunohistochemical analysis of ileal sections from infected mice showed a substantial reduction in macrophages. These results suggested that through the CCL2/CCR2 pathway, infected neurons recruited macrophages to infiltrate the LMMP, which produced proinflammatory factors or secreted soluble factors. Macrophages directly or indirectly destroyed the integrity of peripheral nerves while exerting a viral clearance effect, leading to neuromuscular disorders [11, 51].

However, it has been reported that there is a 2-step HSV-1 vaccination protocol, with an initial intranasal instillation followed by intragastric administration of virus particles, that results in enhanced GI motility and significant acceleration of transit at 1, 2, and 6 weeks after intragastric inoculation with HSV-1. Moreover, experiments have shown the presence of intestinal cholinergic nerve dysfunction during viral infection. Although contrary to the diminished motor frequency described above, viral infection does cause gut dyskinesia [52]. We speculate that the ultimate manifestation of dyskinesia is likely to be a superimposed effect of the ENS and cholinergic nerves.

### Infected neurons express viral antigens to recruit lymphocytes

Unlike the previous study, Brun et al. described a study in which neurons infected with HSV-1 expressed viral antigens, leading them to recruit activated ${CD3}^{+}{CD8}^{+}$ lymphocytes. The mucosal barrier was compromised by the overactivated immune response [29, 53]. In this study, an animal model was established with initial intranasal and subsequent transgastric inoculation of HSV-1. This study elucidated that persistent HSV-1 infection of the ENS can cause fluctuating episodes of GI dyskinesia by continuously observing the frequency of intestinal motility in sacrificed mice at weeks 4, 8, and 10 post transgastric HSV-1 administration. The analysis of the LMMP by flow cytometry and immunohistochemical staining revealed a significant increase in ${CD3}^{+}$ cells from the 6th week of infection after transgastric inoculation with the virus. ${CD8}^{+}$ T cells expressed IL-17, and the expression of IFN-γ in ${CD3}^{+}{CD8}^{+}$ lymphocytes infiltrating the LMMP of mice increased at 6 and 8 weeks after transgastric HSV-1 inoculation. The percentage of ${CD3}^{+}{CD8}^{+}$ cells expressing perforin increased significantly 6 and 8 weeks after intragastric inoculation of HSV-1, while the percentage of ${CD8}^{+}$ cells expressing granzyme B increased 6 weeks after intragastric inoculation. It was also demonstrated that T lymphocytes infiltrating the LMMP tended to have an HSV-1 activated phenotype. Anti-CD8 monoclonal antibody treatment to reduce the number of intestinal ${CD3}^{+}{CD8}^{+}$ cells ameliorated neuronal damage and intestinal dysmotility in HSV-1-infected mice [29, 52, 54]. In addition, acute ENS injury has been shown to result mainly from direct neuronal targeting by antiviral T cells [55].

Motility disorders also occur when the virus invades the esophageal nerves, as is the case for achalasia, which is characterized by impaired relaxation of the lower esophageal sphincter (LES) after swallowing and ineffective esophageal peristalsis [56]. With the COVID-19 pandemic, there has been a report of achalasia after COVID-19 [57]. There have been controlled studies showing an association between the virus and achalasia [58]. Robertson et al. measured statistically significant differences in VZV when conducting serological studies and DNA detection in esophageal tissue sections. [59]. Naik et al. found that cells enriched in immunoreactivity for the late VZV protein gE also present antibodies to neuronal markers (β3-tubulin). This proved that esophageal neurons are the site of VZV infection in achalasia [12]. At present, the mechanism by which HSV-1 causes achalasia is not fully understood, but there is evidence that achalasia may be an immune-mediated inflammatory disease in which the virus is a trigger [56, 60–62]. Facco et al. performed flow cytometry analysis of achalasia versus control esophageal tissue and demonstrated that lymphocyte infiltration was significantly elevated in the LES in achalasia and was represented by${CD3}^{+}{CD8}^{+}$T cells. Lymphocytes extracted from the esophagus of achalasia patients were highly reactive to HSV-1 antigen, with a skewed TCR sequence. After incubation with UV-inactivated HSV-1, IFN-γ and IL-2 were significantly released. Therefore, achalasia should be considered a motor disorder caused by neuronal damage of inflammatory lymphocyte infiltration induced by HSV-1 in the LES [56].

### Neutrophil involvement in GI motility disorders

Khoury-Hanold et al. indicated a role of neutrophils in viral infections in the intestine. When HSV-1 infects the ENS, viral genes begin transcription and replication, which leads to the induction of a large number of inflammatory chemokines, including neutrophils, into the myenteric layer of the large intestine and the destruction of the enteric ganglia [63]. The depletion of neutrophils with an anti-Ly6G (1A8) antibody had little effect on viral replication. However, neutrophil depletion reduced the mortality due to intestinal dyskinesia by one-fifth, and intestinal dyskinesia recovered over time in surviving mice. ENS imaging by whole-mount microscopy showed that the ENS ganglia of the control mice were eliminated during HSV-1 infection, whereas the ENS of the experimental group, that is, neutrophil-depleted mice, remained intact. The study by Khoury-Hanold et al. concluded that HSV-1 infection of the ENS caused neutrophil-mediated neuronal damage, which in turn led to toxic megacolon [40].

### Regulation of gut microbiota

COVID-19 has many extrapulmonary manifestations. GI symptoms associated with COVID-19 are mostly mild and self-limiting and include nausea, vomiting, abdominal pain and abdominal discomfort [46]. In addition to the recruitment of various inflammatory factors, such as IL-2, IL-7, and ΤΝF-α, during viral infection and the possible direct tissue damage caused by viral replication, it has been reported that intestinal inflammation may be mainly regulated by ACE2 receptor-mediated intestinal microbiota [64–66]. This can lead to an imbalance in the proportion of gut flora [67]; for example, the proportion of GI infections caused by streptococci and clostridial organisms will be relatively increased. In addition, due to the role of ACE2 in maintaining amino acid homeostasis, the serum concentration of some amino acids, such as valine and tryptophan, may be significantly decreased when the ACE2 receptor is decreased after SARS-CoV-2 infection [22, 47]. Intestinal microbial disorders might regulate the immune response and affect the progression of diseases [68].

### HIV Tat promotes the LPS-TLR4 signaling pathway and neuronal excitability

Currently, the literature suggests that HIV damages neurons indirectly by releasing virions, such as transactivator of transcription (Tat) [19]. Guedia et al. found that HIV Tat released from HIV-infected cells causes GI dysfunction by promoting the LPS-TLR4 signaling pathway [69, 70]. First, according to studies on ${Tat}^{+}$ mice and ${Tat}^{-}$ mice, enteric neuron/glial cell coculture in the former led to increased release of pro-inflammatory factors such as RANTES, IL-6 [19], IL-1β and TNF-α mRNA. Second, the sensitizing effect of Tat on LPS was not reflected by knocking down TLR4 or treating MyD88 with siRNA. Thus, TLR4 is the major receptor of LPS. When Tat binds to the TLR4 receptor, it can increase the activation of the LPS-TLR4-MyD88-NF-κB pathway, thereby increasing the synthesis and release of proinflammatory factors such as TNF-α, IL-6 and IL-1β [71] (Fig. 3C). The increase in inflammation can significantly destroy GI motility [35]. Second, Ngwainmbi et al. also demonstrated that Tat exposure increased enteric neuronal excitability and thus enhanced GI motility through current-clamp experiments. This may be due to the activation of sodium channels and the increased expression of the sodium channel isoforms Nav1.7 and Nav1.8 [19, 72].

Braun et al. found that neurotropic flaviviruses had a strong ability to infect the ENS. They revealed two major steps by which viruses cause intestinal dysmotility. First, viral invasion led to acute viral infection, which induced neuronal depletion mediated by ${CD8}^{+}$T cells and associated dysmotility [73]. Second, the depleted ENS produced an inflammatory stimulus response under the action of inflammatory mediators and caused recurrent motility disorders [55].

## Conclusion

Viral infection is an important cause of GI motility disorders. In the GI tract, viral infection can lead to nerve damage, which in turn can affect the function of the GI tract.

### Clinical manifestations of intestinal motility disorders after viral infection

Intestinal motility caused by viral infections can have different clinical manifestations depending on the site of the disorder. Achalasia, for example, is a kind of esophageal movement disorder that can cause difficulty swallowing, reflux, heartburn, weight loss and other clinical manifestations. In most studies, the average age at diagnosis was over 50 years, and the incidence increased with age. There was no significant difference in population prevalence. The clinical manifestations of gastroesophageal reflux disease are mainly reflux and heartburn, and the manifestations pseudointestinal obstruction are mainly abdominal pain, bloating, nausea and vomiting.

### Viral infection causes nerve damage through a variety of mechanisms

On the one hand, viruses can directly infect nerve cells, leading to nerve cell death and dysfunction. On the other hand, viral infection can cause an inflammatory response, leading to the release of a large number of inflammatory factors and cytokines, which in turn leads to nerve cell damage and death. In addition, viral infection can cause inflammatory responses in neurons, leading to autoimmune responses, which in turn lead to nerve cell damage and death. Therefore, future research on the mechanism of viral neurological damage in GI motility diseases can be carried out from the following aspects:

#### Studies of the mechanism of the neuronal inflammatory response caused by viral infection

Viral infection can lead to an inflammatory response in neurons, resulting in an autoimmune response, which then results in nerve cell damage and death. Future studies can explore the mechanism of the neuronal inflammatory response caused by viral infection and find targeted treatments.

#### Studies of the mechanism of neuronal death caused by viral infection

Viruses can infect nerve cells directly, resulting in cell death and dysfunction. Future studies can explore the mechanism of neuronal death caused by viral infection and find targeted treatments.

#### Studies of the mechanism of GI inflammation caused by viral infection

When infected with a virus, the body can produce an inflammatory response, leading to the release of a large amount of inflammatory factors and cytokines. This can cause nerve cell damage and death. Future studies can explore the mechanism of GI inflammation caused by viral infection and find targeted treatment methods.

#### Studies of the interaction between viruses and the intestinal microbial community

The intestinal microbial community is one of the key factors for GI health. Viral infection may affect the balance of the intestinal microbial community. Future studies can explore the interaction between viruses and gut microbiota and find targeted treatments.

### Antiviral therapy is of great significance for the treatment of GI diseases

Viral infection causes an inflammatory response in the GI tract, resulting in neuronal damage and dysfunction. Viruses can affect the nervous system of the GI tract through a variety of pathways, including by directly infecting neurons, causing an inflammatory response, and by affecting the synthesis and release of neurotransmitters. Therefore, antiviral therapy is vital for the treatment of GI motility disorders.

Antiviral therapy can inhibit viral replication and infection and reduce the inflammatory response and neuronal damage of the GI tract, thereby alleviating the condition and symptoms. Antiviral therapy can effectively control the progression of GI motility disorders, reduce the pain and discomfort of patients, and improve the quality of life and health of patients.

In addition, antiviral therapy can prevent the occurrence of GI motility disorders. GI motility disorders are closely related to viral infection. Antiviral therapy can protect the health and life of patients by reducing the spread and infection of the virus and preventing GI motility disease.

In conclusion, the mechanism of neurological damage by viruses in GI motility disorders is a complex issue that needs to be investigated by integrating multiple factors. Future research can find targeted treatment methods from different perspectives and provide new ideas and methods for the treatment of diseases. Moreover, antiviral therapy is significant for the treatment and prevention of disorders. In clinical practice, we should pay attention to the role of antiviral therapy in GI diseases and actively carry out relevant research and clinical applications to provide better medical services and health protection for patients.

## Data Availability

Not applicable.

## Abbreviations

GI: gastrointestinal
ENS: enteric nervous system
HSV: herpes simplex virus
VZV: varicella-zoster virus
DRG: dorsal root ganglion
ADV: Aujeszky’s disease virus
CNS: central nervous system
BDV: Borna disease virus
CALB: Calbindin D-28k
PBMCs: peripheral blood mononuclear cells
HELF: human embryonic lung fibroblasts
RTX: resiniferatoxin
LMMP: longitudinal myenteric plexus
TRPV1: transient receptor potential cation channel subfamily V member 1
LES: lower esophageal sphincter
Tat: transcription
